# A Real-World Benchmark for Sentinel-2 Multi-Image Super-Resolution

**DOI:** 10.1038/s41597-023-02538-9

**Published:** 2023-09-21

**Authors:** Pawel Kowaleczko, Tomasz Tarasiewicz, Maciej Ziaja, Daniel Kostrzewa, Jakub Nalepa, Przemyslaw Rokita, Michal Kawulok

**Affiliations:** 1 KP Labs, Gliwice, Poland; 2 Warsaw University of Technology, Warsaw, Poland; 3 Silesian University of Technology, Faculty of Automatic Control, Electronics and Computer Science, Gliwice, Poland

**Keywords:** Computer science, Software, Information technology

## Abstract

Insufficient image spatial resolution is a serious limitation in many practical scenarios, especially when acquiring images at a finer scale is infeasible or brings higher costs. This is inherent to remote sensing, including Sentinel-2 satellite images that are available free of charge at a high revisit frequency, but whose spatial resolution is limited to 10m ground sampling distance. The resolution can be increased with super-resolution algorithms, in particular when performed from multiple images captured at subsequent revisits of a satellite, taking advantage of information fusion that leads to enhanced reconstruction accuracy. One of the obstacles in multi-image super-resolution consists in the scarcity of real-world benchmarks—commonly, simulated data are exploited which do not fully reflect the operating conditions. In this paper, we introduce a new benchmark (named MuS2) for super-resolving multiple Sentinel-2 images, with WorldView-2 imagery used as the high-resolution reference. Within MuS2, we publish the first end-to-end evaluation procedure for this problem which we expect to help the researchers in advancing the state of the art in multi-image super-resolution.

## Background & Summary

Super-resolution (SR) is aimed at reconstructing a high-resolution (HR) image from a single image or multiple low-resolution (LR) observations presenting the same scene. Multi-image SR (MISR) fuses multiple LR images, each of which contains a different portion of HR information. This allows for achieving higher reconstruction accuracy than relying on single-image SR (SISR)^1^, but MISR is highly sensitive to the variability of the input images and their proper co-registration^2^. This poses a challenge when preparing the data for training and validation. Recent advances in satellite image SR include SISR^3^ and MISR^4^ techniques for enhancing Sentinel-2 (S-2) multispectral images (MSIs), composed of 13 bands, whose resolution ranges from 60 m ground sampling distance (GSD) to 10 m GSD^5^.

Commonly, SR techniques are evaluated relying on an artificial scenario–a certain image is treated as an HR reference which is subsequently degraded to obtain the simulated LR images^6–8^. The similarity of the super-resolved outcome to the reference is then used to evaluate the SR performance. Unfortunately, such procedure does not reflect the real-world operating conditions^9^, and methods that perform well for the simulated data are not necessarily effective for original (i.e., not downsampled) images. It is therefore crucial to properly validate the emerging techniques using real LR images coupled with a real HR reference–in an excellent survey on real-world SISR^9^, Chen *et al*. identified the deficiency of realistic datasets as one of the most important challenges in this field. Recently, several real-world SISR datasets have been elaborated^10–12^, however preparing such datasets for MISR is much more costly and troublesome.

In 2019, European Space Agency organized an SR challenge^13^ based on real-world scenes acquired by the PROBA-V satellite, each of which contains an HR image (100 m GSD) coupled with at least nine LR images (300 m GSD). The dataset allowed for developing first MISR techniques underpinned with convolutional neural networks (CNNs), applied either to enhance the LR images before their multi-temporal fusion^14^ or employed to learn the reconstruction process in an end-to-end manner^2,15,16^.Very recently, the WorldStrat dataset was published which matches multiple S-2 images with SPOT spectral bands of 6 m GSD^17^ (the magnification factor equals 1.67×). However, the problem of comparing the reconstruction outcome with the reference was not discussed there and it is not clear whether and how WorldStrat can be used for benchmarking SR algorithms. We demonstrate that this is not a straightforward task, especially when LR and HR images are captured by different satellites. In^18^, S-2 SISR was assessed by comparing the outcome against WorldView-3 images. The authors observed that the peak signal-to-noise ratio (PSNR) and structural similarity index (SSIM) do not correlate well with the quality of the reconstructed images–the highest scores were obtained for blurred images in which the details were not reconstructed accurately. Contrary to that, the learned perceptual image patch similarity (LPIPS)^19^ was reported to be suitable for evaluating SISR for remote sensing^20,21^, but it was not applied to MISR.

In this paper, we address the problem of evaluating MISR for S-2 images by introducing a new Multi-image Sentinel-2 SR (MuS2) benchmark. It is composed of a new dataset^22^ with WorldView-2 (WV-2) images used as an HR reference and the end-to-end validation procedure. Our contribution can be summarized in the following points.

**Fig. 1:**
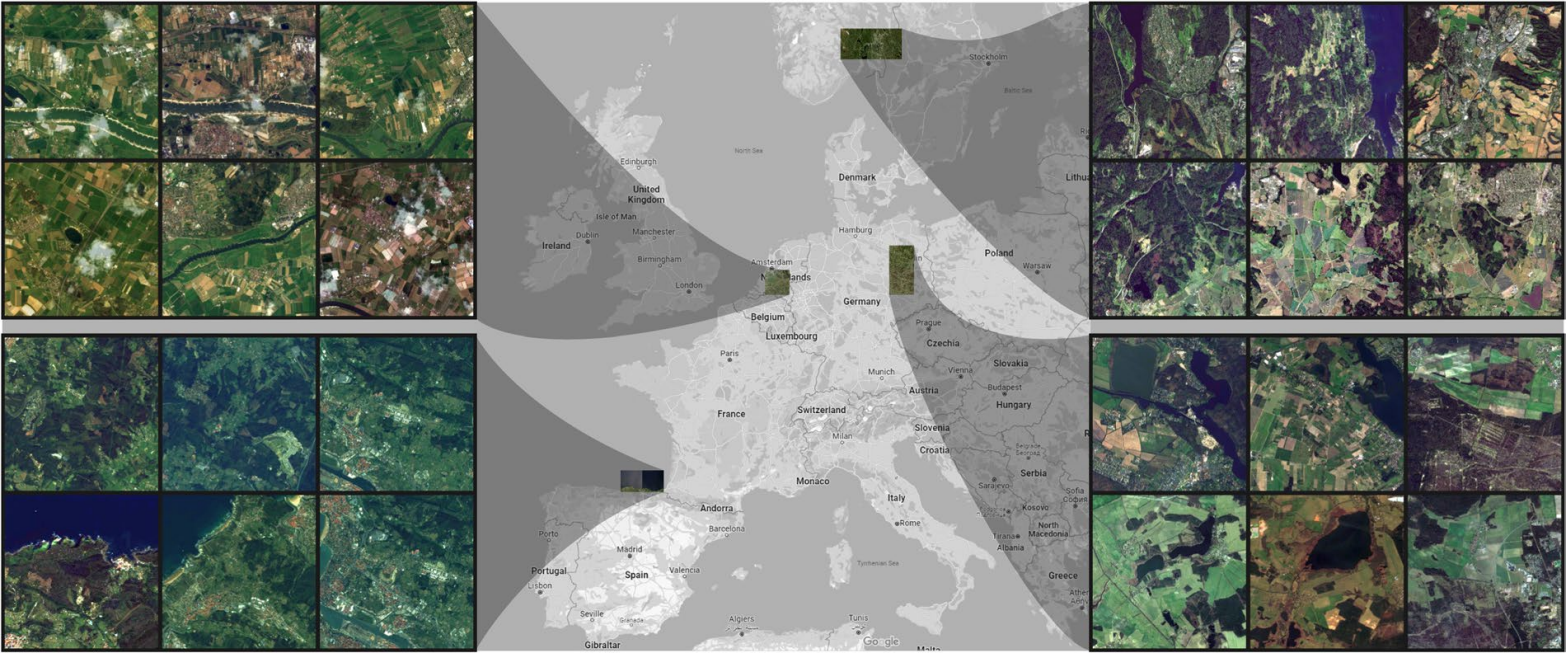
Regions selected for our MuS2 benchmark. The locations of seven S-2 tiles (100 × 100 km each) are shown on the map and six examples of cropped WV-2 images (5 × 5 km each) that are used as HR reference, are presented for each region, i.e., Netherlands (top left), Norway (top right), Spain (bottom left), and Germany (bottom right). The WV-2 examples were cropped from the original scenes (up to 6.5 × 6.5 km) to demonstrate the diversity of the regions included in the benchmark. Map source: Google.
- We elaborated a protocol (included into the source code we publish) for assessing the SR outcome for all of S-2 10 m bands based on the corresponding WV-2 bands, and we report the results of 3× magnification obtained using the well-established MISR techniques.
- We demonstrate that the LPIPS metric^19^ is suitable for evaluating the MISR outcome, even if the reference HR images are acquired using a different satellite. To verify that, we have performed a mean opinion score (MOS) survey, whose results are thoroughly discussed.
- We introduce change masks coupled with relevance masks which indicate the image regions that can be considered for evaluation relying on pixel-wise image similarity metrics.

- We publish a new MuS2 benchmark dataset^22^ with 91 diverse scenes covering over 3200 km^2^ (Fig. 1), each composed of at least 14 S-2 MSIs coupled with a single WV-2 MSI. The benchmark is intended to serve as a test set for evaluating future advancements in MISR.

## Methods

### Data source

Our benchmark dataset^22^ is composed of images acquired by S-2 and WV-2 satellites. The S-2 images were downloaded from the Copernicus Open Access Hub, and we accessed the WV-2 images published within the European Cities dataset (available at https://earth.esa.int/web/guest/-/worldview-2-european-cities-dataset). The S-2 data are organized into tiles, each of which contains a 100 × 100 km MSI, and aggregated into products that are distributed at different processing levels. Here, we exploit Level-2A which includes the bottom of atmosphere reflectance correction. Each tile is composed of 13 spectral bands, however the B10 (cirrus) band is excluded from Level-2A, because it does not represent any surface information. The blue, green, red, and near infrared (NIR) bands (B02, B03, B04, and B08, respectively) are of 10 m GSD, the vegetation red edge bands (B05, B06, and B07), narrow NIR (B08a), and the short-wave infrared (SWIR) bands (B11 and B12) are of 20 m GSD, while the remaining coastal aerosol (B01), water vapour (B09), and cirrus clouds estimation (B10) bands are of 60 m GSD.

Each WV-2 tile contains a panchromatic image of 0.4 m GSD and 8 spectral bands at 1.6 m GSD. These are C–coastal (400–450 nm), B–blue (450–510 nm), G–green (510–580 nm), Y–yellow (585–625 nm), R–red (630–690 nm), RE–red edge (705–745 nm), NIR1 (770–895 nm), and NIR2 (860–1040 nm). Based on the coverage between the spectral ranges of S-2 and WV-2 bands (measured with the Dice coefficient–*D*_*C*_), we have selected four pairs: B02 with B (*D*_*C*_ = 0.8), B03 with G (*D*_*C*_ = 0.68), B04 with R (*D*_*C*_ = 0.68), and B08 with NIR1 (*D*_*C*_ = 0.92). For the remaining band pairs, the spectral coverage did not exceed *D*_*C*_ = 0.5, so we do not consider them in our study.

### Scene selection and data alignment

The scenes for our MuS2 benchmark were selected from the European Cities dataset so as to ensure high landcover diversity, and then we searched for multiple corresponding S-2 images for each scene–we aimed at collecting around 15 LR images of high quality, with none or low cloud coverage, for every HR WV-2 reference. The number of LR images was chosen based on the results reported in various works on MISR^15,16,23,24^–initially, the reconstruction outcome is improved with an increasing number of input LR images presenting the same scene, but this growth stabilizes at around 10–12 input LR images (and it was even reported to drop afterwards for the PIUnet method^15^). Some areas had to be rejected due to insufficient data availability (often because of poor quality resulting from cloud coverage and atmospheric conditions), and eventually we prepared 91 WV-2 tiles, each covering a square of 6.5 × 6.5 km, that fall into seven different military grid reference system (MGRS) tiles located in Netherlands, Norway, Spain, and Germany (see Fig. 1). They present various terrain and landcover types, including urban areas, plains, cultivated fields, mountains, and coastal areas. Finally, for 48 scenes, we have gathered 15 LR images, and for 43 scenes–14 LR images.

**Fig. 2:**
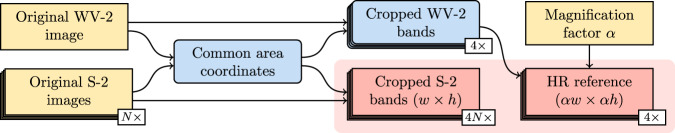
The data preparation pipeline: the yellow boxes indicate input data, the blue ones–temporary data, and the red ones–the output. For each WV-2 image, *N* S-2 images with overlapping area are processed to identify the coordinates of the largest common region. It is used to crop the four 10 m bands from S-2 images and corresponding WV-2 bands. The latter are downsampled, so that they are *α*× larger than the cropped S-2 images.

The collected scenes were subsequently subject to our data preparation procedure (see Fig. 2) which operates on original S-2 and WV-2 tiles. The common area is determined based on the geographic coordinates retrieved from the metadata, and for each band, *N* LR images (II_in_) are cropped and coupled with the WV-2 image (which in some cases is also cropped beforehand to remove blank pixels in the corners that do not contain any data). Each resulting WV-2 image is subsequently downsampled to create the HR reference (II_HR_) whose each dimension is *α*× larger than those of II_in_. In the published dataset^22^, we use *α* = 3, as enlarging the images 3× remains sufficiently challenging for the state-of-the-art MISR techniques^15,16,25,26^. However, the elaborated software tools that we publish along with the benchmark allow for generating the reference HR data up to *α* = 6.25 for WV-2 images of the original size.

**Fig. 3:**
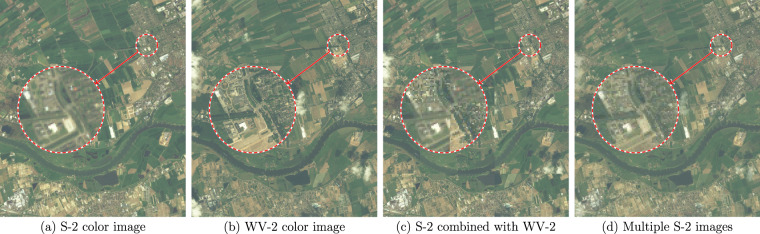
Color images composed of S-2 and WV-2 bands to inspect the co-registration correctness of the prepared data. The example shows correct co-registration among (**a**) S-2 and (**b**) WV-2 bands, (**c**) between S-2 and WV-2 images (in a form of a checkerboard image), and (**d**) among multiple S-2 images (incorrect co-registration would result in color artefacts). The images present an area of 5.5 × 6.5 km near Wageningen, Netherlands–the WV-2 image was acquired in June 2011, and S-2 images were acquired in April 2019–March 2021.

Overall, we acquired 91 HR scenes, whose characteristics are summarized in Table 1. Every HR and LR image contains all the spectral bands (including the aforementioned four matched WV-2–S-2 band pairs), and they are also accompanied with a panchromatic image for WV-2. To verify whether the bands are correctly co-registered after cropping, we composed color images and inspected them against color artefacts (Fig. 3a,b). In order to check the co-registration correctness between LR images and the HR image, we have assembled checkerboard mosaicked images and inspected them visually for all the scenes (Fig. 3c). Finally, we combined multiple S-2 images for each scene to inspect whether the obtained images are free of color and halo artefacts that could result from poor co-registration across multiple S-2 images and/or the spectral bands (Fig. 3d).

**Table 1:** Characteristics of the MuS2 benchmark^22^.

Parameter	Value (or range)
Number of scenes	91
LR images per scene	14 (43 scenes) or 15 (48 scenes)
Scene height	3.3–6.5 km
Scene width	4.0–6.5 km
Area (per scene)	20.4–42.9 km^2^
Average scene area	35.3 km^2^
Total area	3207.5 km^2^
WV-2 acquisition date	September 2010–March 2015
S-2 acquisition date	April 2019–March 2021

### The evaluation procedure

The evaluated SR process is fed with *N* input images for reconstructing a specific S-2 band to produce a single super-resolved image (II_SR_) which is subsequently compared with the corresponding II_HR_. By an input image we understand a single S-2 band, but it is also possible that multiple bands cropped to show the same area are processed to reconstruct a single S-2 band or multiple bands at once^24^. Afterwards, each of the super-resolved 10 m bands can be compared with a corresponding II_HR_ derived from the WV-2 data. We measure the similarity between II_SR_ and II_HR_ with the PSNR, SSIM, and LPIPS metrics that are commonly employed for assessing the SR quality^20^. II_in_ and II_HR_ are acquired by different sensors, hence the differences between II_SR_ and II_HR_ result not only from the lack of reconstruction accuracy, but also from the characteristics of the imaging sensor and the temporal changes of the scene (due to the difference in the acquisition time)^27^. Also, as the *N* input images are co-registered in the LR space at whole-pixel precision, the super-resolved II_SR_ may be displaced up to *α* pixels in each dimension compared with II_HR_. For PROBA-V images (acquired with the same satellite), it was sufficient to co-register II_SR_ to II_HR_ and to compensate the brightness bias^13^. The co-registration was performed by shifting II_SR_ by [-*α*; *α*] pixels in vertical and horizontal dimensions to maximize the PSNR score computed for the whole II_HR_ image without an *α*-wide boundary (so that even after shifting the same part of II_HR_ is considered for computing the score). For MuS2, we have fully adopted that approach. In^13^, the brightness was compensated by subtracting the mean brightness from both II_SR_ and II_HR_. In the case considered here, the differences between S-2 and WV-2 images are more substantial than among PROBA-V images, therefore we match the histogram of II_SR_ to II_HR_ before the evaluation. Importantly, this does not convey any HR information from II_HR_ to II_SR_, but only compensates the low-frequency differences between them. Also, we do not modify II_HR_, so that every II_SR_ obtained using different techniques is always compared to the same reference image.

As proposed in^13^, in addition to reporting the direct values of PSNR, we also compute their relation to the scores retrieved for images obtained by averaging LR images enlarged with bicubic interpolation, treated as a baseline which does not offer any information gain. We adopt the same approach for SSIM and LPIPS metrics, and we also compute the balanced metric as:

1 $${\mathscr{B}}\left({{\mathbb{I}}}_{{\rm{SR}}}\right)=\frac{1}{3}\left[\frac{{\rm{PSNR}}\left({{\mathbb{I}}}_{{\rm{bic}}},{{\mathbb{I}}}_{{\rm{HR}}}\right)}{{\rm{PSNR}}\left({{\mathbb{I}}}_{{\rm{SR}}},{{\mathbb{I}}}_{{\rm{HR}}}\right)}+\frac{{\rm{SSIM}}\left({{\mathbb{I}}}_{{\rm{bic}}},{{\mathbb{I}}}_{{\rm{HR}}}\right)}{{\rm{SSIM}}\left({{\mathbb{I}}}_{{\rm{SR}}},{{\mathbb{I}}}_{{\rm{HR}}}\right)}+\frac{{\rm{LPIPS}}\left({{\mathbb{I}}}_{{\rm{SR}}},{{\mathbb{I}}}_{{\rm{HR}}}\right)}{{\rm{LPIPS}}\left({{\mathbb{I}}}_{{\rm{bic}}},{{\mathbb{I}}}_{{\rm{HR}}}\right)}\right],$$

where II_bic_ is obtained by averaging all bicubically-upsampled II_in_’s in the scene (they are all co-registered as justified earlier). Hence, ${\mathscr{B}}$ < 1 means better performance compared with the bicubic interpolation, and ${\mathscr{B}}$ > 1 indicates the opposite case (for PSNR and SSIM the higher score indicates higher similarity, and for LPIPS the lower the score, the higher the similarity).

**Fig. 4:**
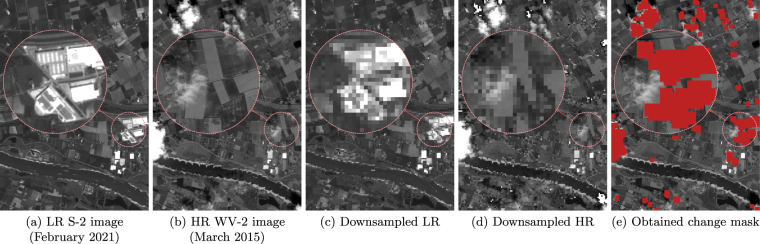
An example of creating the change mask between S-2 and WV-2 images. New structures were erected between the years 2015 and 2021 (**a,b**), which can be detected from the difference between downsampled LR and HR images (**c,d**) that do not contain high-frequency information. The areas covered by the change mask, marked in red color in (**e**), are not used for assessing the reconstruction quality. For the presented case, 10.7% of the whole image area is excluded by the mask–it captures the erected structures, as well as the clouds that can be seen in the HR WV-2 image. The images present an area of 4.6 × 6.5 km around Valburg, Netherlands. The WV-2 image (the G band is shown here) was acquired in March 2015, and S-2 image (B03 band is presented) in February 2021.

As shown in Table 1, the S-2 images were acquired 4–11 years after their WV-2 counterparts. In order to compensate for the occurring temporal differences, we decided to exclude the areas which were substantially changed between the acquisition dates of WV-2 image and the most recent S-2 image in the series. To detect the changed areas, we applied a fairly simple technique–after matching the LR image histogram to that of the HR reference, both images are downsampled (by block averaging) to the same dimensions in order to remove high-frequency information (LR images are downsampled by a factor of 4× and HR references by a factor of 12×). Afterwards, we compute a map of pixel-wise relative differences between the downsampled images, which is binarized and subjected to morphological opening operation to remove some isolated false positives. This creates a change mask which can be used during evaluation to exclude the pixels, whose HR reference may not be correct. In Fig. 4, we show an example of a changed area–clearly, new structures have been erected since the WV-2 image was acquired, and the change mask excludes that region quite accurately. In addition to that, the areas occluded with clouds in the HR WV-2 image are also masked out–the pixels marked as red in Fig. 4e are ignored when computing the similarity metrics with the change mask applied. Although the problem of change detection has been studied intensively and the state-of-the-art techniques underpinned with deep neural networks allow for obtaining precise segmentation outcome, most of them are limited to processing images acquired by the same sensor^28^, including S-2^29^ and WV-2^30^. Based on visual inspection, we found this simple technique to deliver satisfactory results for the images acquired using different sensors, and it excludes the pixels which should not be considered for evaluation. The code for creating these masks is included into the published package (see Code availability, below).

**Fig. 5:**
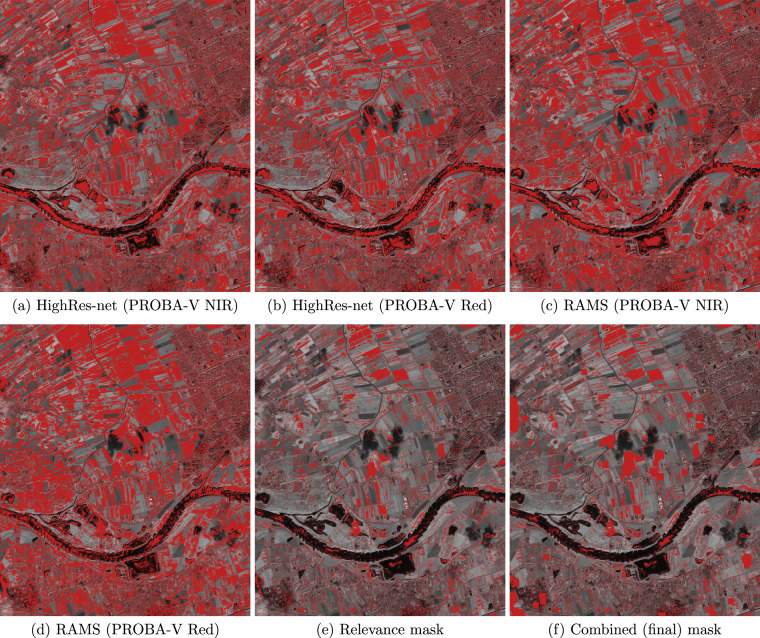
Examples of relevance masks (**a**–**e**) and the final combined mask (**f**) which show the areas excluded from evaluation (marked in red color). The resulting relevance mask (**e**) is built of the pixels, whose values retrieved with all four models trained from non-simulated data (HighRes-net^25^ and RAMS^26^, trained over PROBA-V NIR and Red images, (**a**–**d**)) are less similar to the HR reference than the values in the bicubically-interpolated image. Finally, in (**f**), we show the relevance mask combined with the change mask which excludes the areas that differ much between WV-2 and the latest S-2 image in the temporal series (in this case, it eliminated the clouds located in the center and on the left side). The relevance masks are created independently for each band pair (here, B08/NIR1), whereas the change mask is common for all the bands. The underlying WV-2 image (NIR1) was acquired in June 2011, and it presents an area of 5.5 × 6.5 km near Wageningen, Netherlands.

During our experiments (reported later in this paper), we observed that the PSNR and SSIM metrics are not particularly effective in assessing the reconstruction accuracy when II_in_ and II_HR_ originate from different satellites–the scores differ little across II_SR_’s obtained with the employed SR or interpolation procedures (with and without using the change masks). Based on experimental validation and MOS survey, whose results are discussed later in this paper, it is clear that the outcomes obtained using MISR techniques better reflect the image details than relying on bicubic interpolation, and this is correctly captured relying on the LPIPS metric. It can be seen from Fig. 5 that there are regions, marked as red in Fig. 5e, in which bicubic interpolation consistently prevails over all of the four considered SR techniques trained from real-life data–this is measured relying on the mean squared error (MSE) that the PSNR metric is based on (these regions were extracted after applying shift and brightness compensation). This may result from the temporal differences between II_in_’s and II_HR_, as well as among the II_in_’s themselves–in the regions where bicubic interpolation renders lower MSE than all the SR techniques, the HR reference is regarded as inappropriate to assess the reconstruction quality. It can be noticed that such regions are mainly located in the cultivated fields, while in the highly-detailed urban areas they are isolated (this may result from shadows and differences in lighting conditions). Overall, for every scene, we generate the relevance masks which extract the regions in II_HR_ where at least one MISR model (out of four considered in our study) offers better performance than the bicubic interpolation in terms of MSE, and if the relevance masks are applied, then the metrics are computed only inside such regions. Eventually, these masks are combined with the change masks as shown in Fig. 5f (the pixels marked as red are excluded from the evaluation).

### Discussion and outlook

The proposed evaluation is exclusively focused on reference-based assessment using original HR images. Although some reference-free metrics, like natural image quality evaluator (NIQE)^31^, perception-based image quality evaluator (PIQE)^32^ or no-reference quality metric (NRQM)^33^, are also exploited for scoring real-world SR^9,34^, they primarily assess the quality of a reconstructed image rather than accuracy of reconstructed information. Furthermore, the consistency between the input LR images and the super-resolved output can also be verified, especially for multispectral and hyperspectral data, to ensure that the upsampling process has not affected their spectral characteristics^24^, and it can also be embedded into the loss function during training^4^. Such consistency is commonly verified by downsampling the super-resolved image back to the original dimensions to determine the spectral angle between the original and super-resolved image. As WV-2 images have different spectral properties than S-2 images (which is compensated by histogram matching in MuS2 benchmark protocol), we use the WV-2 images as HR reference to verify the geometric features of the reconstructed details, and the consistency check may also be treated as a complementary validation step. However, as such evaluation may be performed for any set of original S-2 images, we do not include neither the consistency-based, nor reference-free metrics in our study.

Although we have shown that all of the S-2 10 m bands can be roughly coupled with corresponding WV-2 bands to assess the reconstruction accuracy, evaluating reconstruction of the remaining bands, potentially relying on panchromatic WV-2 images, remains an open issue. With our data preparation procedure, it is possible to crop all the bands which may help address this challenging problem in the future. Furthermore, here we consider the magnification factor of 3×, but with the prepared procedure, other factors may be applied as well. Finally, even though the reconstruction outcomes obtained with the reported techniques are of definitely higher quality than the bicubically-interpolated images (which is further discussed later in Technical Validation), they are still far from the HR reference. We expect that MuS2 used as a test set for assessing emerging SR approaches will guide the researchers toward developing more effective solutions. They may result from improved architectures^16^, better data simulation^35^ and augmentation procedures^36,37^, as well as from new loss functions^38,39^ that would allow for more robust training from real-world data^9^.

## Data Records

**Fig. 6:**
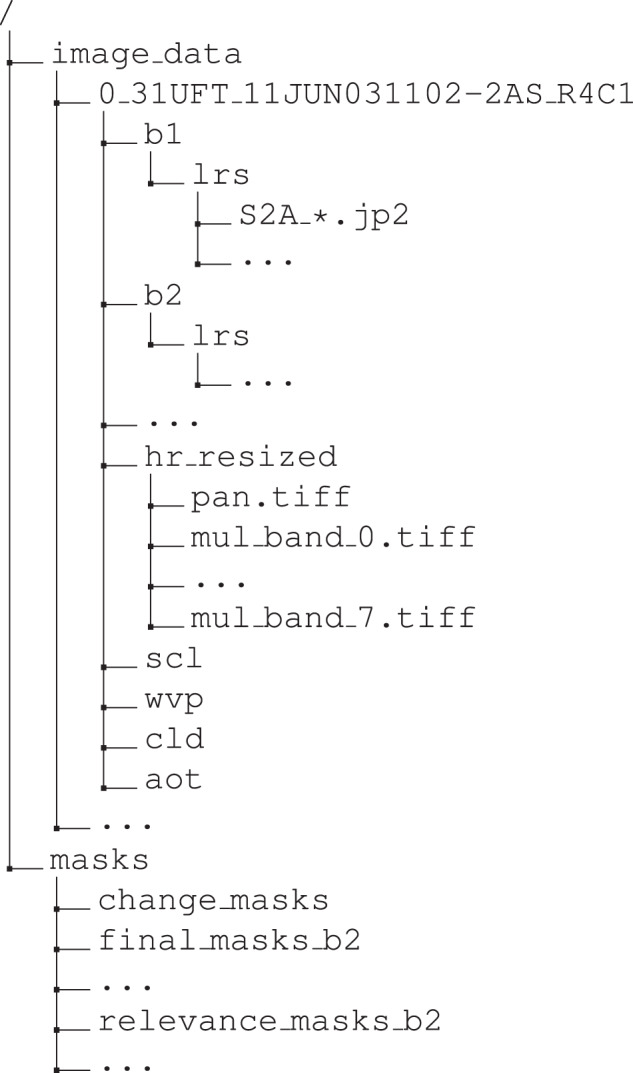
The folder structure of the MuS2 benchmark dataset^22^.

We published the MuS2 benchmark dataset at Harvard Dataverse^22^ and its folder structure is depicted in Fig. 6. The images are grouped into 91 scenes, each of which is placed in a separate scene-level folder, whose names follow the pattern <ord_num>_<mgrs>_<date>-2AS_<tile>, where <ord_num> is the ordinal number of Sentinel-2 product, <mgrs> is the MGRS tile representing the captured area, <date> is the acquisition date of the WV-2 image, and <tile> is the name of the WV-2 tile. Each scene-level folder contains 12 band-level folders (b1, b2, b3, etc.) with the corresponding S-2 bands. Every band-level folder contains 14 or 15 Level-2A images captured at different revisits. The WV-2 bands (numbered from 0 to 7) along with a panchromatic image are stored in the hr_resized folder for each scene, resized to the dimensions 3× larger than the S-2 input images. Also, the scene classification maps (scl), cloud masks (cld), aerosol optical thickness (aot), and water vapour maps (wvp) are included as well. The resolution of HR images is 3× larger than the resolution of S-2 10 m GSD images (i.e., bands B02, B03, B04, and B08). S-2 and WV-2 band images are intended to be coupled for evaluation as follows:

- S-2 B02 (b2) with WV-2 B band (mul_band_1),
- S-2 B03 (b3) with WV-2 G band (mul_band_2),
- S-2 B04 (b4) with WV-2 R band (mul_band_4),
- S-2 B08 (b8) with WV-2 NIR1 band (mul_band_6).

Additionally, the change masks, relevance masks, and the final combined masks for the 10 m S-2 bands are also included in the masks folder.

## Technical Validation

The goal of our experiments was twofold: (*i*) to confirm that MuS2, including the evaluation procedure, is suitable for assessing the reconstruction accuracy, and (*ii*) to report the scores of the well-established SR techniques, which can be used as a baseline for future research. For this sake, we included HighRes-net that recursively combines latent LR representations to obtain the super-resolved image^25^, as well as the residual attention MISR (RAMS) network equipped with the attention mechanism^26^.

In this study, we focus on evaluating MISR performed for four 10 m S-2 bands matched with the HR reference obtained from WV-2. In order to verify that based on MuS2, we can assess how much the reconstructed details resemble those observed in II_HR_, we consider three groups of methods: (*i*) MISR networks trained with real-world LR and HR PROBA-V images^13^, (*ii*) MISR networks trained using S-2 simulated LR data^40^ (obtained by degrading an original S-2 image, later treated as II_HR_), and (*iii*) image interpolation techniques treated as a baseline. In^41^, we demonstrated that RAMS and HighRes-net trained from PROBA-V images are successful in super-resolving S-2 data, and they do not generate artefacts observed when these networks are trained using simulated LR images. To verify how such artefacts influence the scores for MuS2, we report the performance for the networks trained with PROBA-V NIR, PROBA-V Red, and S-2 simulated images.

**Fig. 7:**
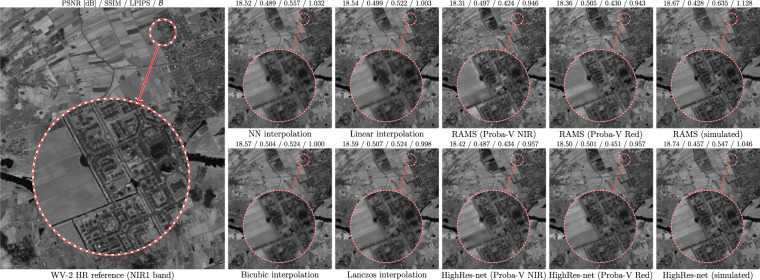
Reconstruction outcome (band B08) obtained with RAMS and HighRes-net trained from real-life PROBA-V NIR and Red images and from simulated data, compared with image interpolation techniques and HR reference. PSNR (in dB), SSIM, LPIPS and ${\mathscr{B}}$ scores are presented above each example. The images present an area of 5.5 × 6.5 km near Wageningen, Netherlands–the WV-2 image was acquired in June 2011, and S-2 images were acquired in April 2019–March 2021.

Quantitative results obtained for four S-2 bands without and with applying the prepared masks are reported in Table 2. It can be seen that without the relevance masks (with or without the change masks), the PSNR and SSIM scores do not differ much between each other within particular bands, and they do not indicate the SR techniques to be better than interpolation. This is against what can be judged from visual inspection of the reconstruction outcome, an example of which is presented in Fig. 7. The images rendered by HighRes-net and RAMS trained over PROBA-V images present more details than the interpolated images (which are quite blurry), and they are free from the artefacts that appear for the models trained from the S-2 simulated data. It is worth noting that based on visual assessment, the details in II_SR_ are quite close to those observed in the WV-2 image, which suggests that they were reconstructed indeed rather than hallucinated (such hallucination artefacts are quite common for SISR techniques, especially for larger magnification factors^42^). However, it can be noticed that these qualitative observations are quantitatively reflected only in the LPIPS values, while PSNR and SSIM are slightly worse for HighRes-net and RAMS–overall, both LPIPS and ${\mathscr{B}}$ indicate that SR networks perform better than interpolation and they penalize for the artefacts. Apparently, the differences between II_in_ and II_HR_ images resulting from different image acquisition conditions prevail over the accuracy of reconstructing the details when local pixel-wise metrics like PSNR or SSIM are used, but they have smaller impact on the feature-based LPIPS metric. As expected, the use of change masks increases the similarity scores, but it does not influence the general tendencies of the scores. However, when the relevance masks are applied (also when they are combined with the change masks), all the metrics indicate the superiority of SR networks and their ${\mathscr{B}}$ scores are aligned in a similar order as without the masks.

**Table 2:** Reconstruction accuracy obtained for our MuS2 benchmark measured with PSNR (in dB), SSIM, LPIPS, and the balanced score ${\mathscr{B}}$, obtained for different interpolation techniques alongside HighRes-net and RAMS networks trained using real-world and simulated images.

Method ↓		Band B02				Band B03				Band B04				Band B08			
		PSNR	SSIM	LPIPS	${\mathscr{B}}$	PSNR	SSIM	LPIPS	${\mathscr{B}}$	PSNR	SSIM	LPIPS	${\mathscr{B}}$	PSNR	SSIM	LPIPS	${\mathscr{B}}$
No mask applied (whole image compared):																	
Interp.	NN	34.85	0.9266	0.2705	0.984	30.66	0.8465	0.3733	0.979	31.80	0.8575	0.3451	0.988	24.06	0.5694	0.5285	1.001
	Linear	34.92	0.**9305**	0.2986	1.016	30.72	0.8533	0.4157	1.012	31.85	0.8627	0.3762	1.015	24.10	0.5790	0.5318	0.998
	Bicubic	34.95	0.9301	0.2858	1.000	30.75	0.8535	0.4025	1.000	31.88	0.8631	0.3609	1.000	24.14	0.5835	0.5419	1.000
	Lanczos	34.95	0.9302	0.2817	0.995	30.76	0.**8540**	0.3974	0.996	**31.89**	0.**8636**	0.3568	0.996	**24.15**	0.5853	0.5408	0.998
HRn	Pr-NIR	**35.19**	0.9288	0.2310	0.936	**30.84**	0.8498	0.3214	0.934	31.82	0.8604	0.2864	0.932	23.93	0.5820	**0.4330**	**0.936**
	Pr-Red	34.95	0.9229	0.2192	0.925	30.68	0.8437	0.3164	0.933	31.61	0.8553	0.2792	0.929	23.98	0.5842	0.4446	0.941
	S2-simul.	34.63	0.9125	0.2730	0.998	30.39	0.8247	0.3610	0.988	31.48	0.8383	0.3412	1.004	23.69	0.5311	0.4901	1.014
RAMS	Pr-NIR	35.01	0.9232	0.2166	0.922	30.61	0.8400	0.3171	0.936	31.59	0.8543	0.2757	0.927	23.97	0.5850	0.4391	0.937
	Pr-Red	35.11	0.9257	**0.2165**	**0.917**	30.80	0.8478	**0.3107**	**0.925**	31.87	0.8605	**0.2706**	**0.915**	24.01	0.**5854**	0.4558	0.947
	S2-simul.	34.55	0.9143	0.2580	0.984	30.35	0.8238	0.3660	0.990	31.51	0.8345	0.3503	1.006	23.84	0.5261	0.5617	1.056
Change masks applied:																	
Interp.	NN	35.57	0.9319	0.2626	0.984	31.33	0.8536	0.3653	0.979	32.37	0.8644	0.3366	0.988	24.19	0.5790	0.5209	1.000
	Linear	35.65	0.9357	0.2904	1.015	31.40	0.8601	0.4074	1.011	32.43	0.8694	0.3673	1.015	24.24	0.5883	0.5250	0.997
	Bicubic	35.67	0.9353	0.2778	1.000	31.43	0.8604	0.3944	1.000	32.46	0.8699	0.3524	1.000	24.28	0.5929	0.5348	1.000
	Lanczos	35.68	0.9355	0.2738	0.995	31.44	**0.8609**	0.3894	0.995	**32.47**	**0.8705**	0.3484	0.996	**24.29**	0.5947	0.5338	0.998
HRn	Pr-NIR	**35.90**	0.9340	0.2240	0.934	**31.49**	0.8568	0.3146	0.933	32.36	0.8673	0.2793	0.933	24.07	0.5918	**0.4275**	**0.937**
	Pr-Red	35.62	0.9279	0.2126	0.925	31.29	0.8506	0.3097	0.934	32.15	0.8622	0.2723	0.930	24.12	0.5937	0.4392	0.942
	S2-simul.	35.34	0.9184	0.2655	0.994	31.05	0.8324	0.3532	0.980	32.04	0.8460	0.3330	0.995	23.81	0.5422	0.4820	1.005
RAMS	Pr-NIR	35.66	0.9281	0.2101	0.921	31.23	0.8471	0.3103	0.936	32.11	0.8611	0.2686	0.928	24.11	**0.5950**	0.4333	0.938
	Pr-Red	35.77	0.9312	**0.2099**	**0.919**	31.42	0.8548	**0.3041**	**0.926**	32.40	0.8674	**0.2638**	**0.918**	24.15	**0.5950**	0.4503	0.948
	S2-simul.	35.22	0.9203	0.2498	0.976	30.98	0.8320	0.3571	0.985	32.06	0.8427	0.3408	1.004	23.97	0.5378	0.5520	1.049
Relevance masks applied:																	
Interp.	NN	34.60	0.9613	0.2226	1.001	30.39	0.9359	0.3021	0.999	31.56	0.9091	0.2710	1.001	24.01	0.8327	0.4149	1.003
	Linear	34.64	0.9628	0.2295	1.011	30.42	0.9381	0.3132	1.010	31.58	0.9114	0.2810	1.013	24.03	0.8360	0.4239	1.009
	Bicubic	34.70	0.9626	0.2225	1.000	30.48	0.9383	0.3049	1.000	31.65	0.9119	0.2716	1.000	24.11	0.8385	0.4156	1.000
	Lanczos	34.71	0.9627	0.2203	0.997	30.49	**0.9384**	0.3023	0.997	31.66	0.9122	0.2694	0.997	24.12	0.8392	0.4119	0.997
HRn	Pr-NIR	**35.41**	**0.9630**	0.2021	0.963	**31.06**	**0.9384**	0.2693	0.955	**32.14**	0.9135	0.2404	0.956	24.40	0.8401	0.3503	0.943
	Pr-Red	35.24	0.9608	0.1987	0.960	30.96	0.9369	0.2667	0.954	31.96	0.9111	0.2375	0.955	24.33	0.8404	0.3626	0.954
	S2-simul.	34.57	0.9555	0.2425	1.034	30.32	0.9272	0.3210	1.023	31.45	0.8983	0.2949	1.036	23.81	0.8152	0.4271	1.023
RAMS	Pr-NIR	35.26	0.9611	0.**1939**	**0.952**	30.88	0.9352	0.2675	0.956	31.89	0.9108	0.2338	0.951	**24.51**	0.8406	**0.3394**	**0.933**
	Pr-Red	35.29	0.9619	0.1953	0.954	30.98	0.9379	**0.2621**	**0.948**	32.11	**0.9144**	**0.2329**	**0.947**	24.47	0.**8411**	0.3567	0.947
	S2-simul.	34.39	0.9544	0.2393	1.031	30.19	0.9241	0.3315	1.037	31.41	0.8951	0.3090	1.055	23.97	0.8122	0.4721	1.058
Combined (final) masks applied:																	
Interp.	NN	36.62	0.9716	0.1943	0.997	32.15	0.9479	0.2749	0.994	32.89	0.9222	0.2461	0.997	24.21	0.8420	0.3992	1.001
	Linear	36.67	**0.9727**	0.2025	1.010	32.19	0.9496	0.2884	1.009	32.91	0.9241	0.2580	1.012	24.22	0.8450	0.4099	1.008
	Bicubic	36.71	0.9726	0.1965	1.000	32.24	0.9498	0.2809	1.000	32.97	0.9246	0.2494	1.000	24.31	0.8475	0.4024	1.000
	Lanczos	36.71	**0.9727**	0.1944	0.997	32.25	**0.9499**	0.2785	0.997	32.98	0.9249	0.2473	0.997	24.32	0.8481	0.3989	0.997
HRn	Pr-NIR	**37.34**	0.9726	0.1778	0.963	32.72	0.9497	0.2455	0.953	**33.37**	0.9254	0.2191	0.955	24.60	0.8494	0.3373	0.941
	Pr-Red	37.12	0.9703	0.1755	0.962	32.58	0.9480	0.2436	0.953	33.19	0.9231	0.2165	0.954	24.53	0.8493	0.3485	0.952
	S2-simul.	36.39	0.9659	0.2175	1.041	31.94	0.9394	0.2960	1.025	32.66	0.9114	0.2715	1.038	24.00	0.8260	0.4107	1.020
RAMS	Pr-NIR	37.14	0.9706	**0.1704**	**0.953**	32.51	0.9466	0.2441	0.955	33.12	0.9230	0.2126	0.950	**24.73**	0.8502	**0.3261**	**0.930**
	Pr-Red	37.16	0.9717	0.1714	0.954	32.57	0.9492	**0.2386**	**0.947**	33.34	**0.9263**	**0.2118**	**0.945**	24.67	**0.8503**	0.3431	0.945
	S2-simul.	36.14	0.9655	0.2122	1.034	31.76	0.9372	0.3043	1.037	32.61	0.9092	0.2833	1.055	24.17	0.8234	0.4529	1.053

The scores are reported without and with using the prepared masks. The best scores (highest PSNR and SSIM, and lowest LPIPS and ${\mathscr{B}}$) are boldfaced.

HRn–HighRes-net, trained with PROBA-V NIR (Pr-NIR), PROBA-V Red (Pr-Red), or S-2 simulated (Simul.) images.

NN–nearest-neighbor interpolation.

As shown in Table 2, the reported metrics (without the relevance masks) are not consistent in indicating the SR performance. In order to verify which of them is most reliable for assessing the reconstruction accuracy, we have prepared a MOS survey composed of 15 queries (the MOS questions are presented in Table S1 in the Supplementary Information). We considered the cases in which PSNR, SSIM, and LPIPS metrics pointed to a different SR or interpolation outcome as the most (eight cases) or the least (seven cases) similar to II_HR_. In each query, the participants of diverse background (including Earth observation professionals and individuals without any remote sensing experience) were presented such three images alongside II_HR_, and asked to select the best or the worst image for retrieving some specific details (e.g., delineating the roads). In this way, we wanted to prevent the participants from being biased toward picking an image of the best perceptual quality, instead of the one whose details are most similar to those in II_HR_. The results of the survey (averaged from over 160 responses) are reported in Table 3–clearly, LPIPS correlates most with the human judgement both in picking the most or least accurate reconstruction outcome (retrieved with an SR network trained from PROBA-V data and an interpolation technique, respectively). Overall, even though LPIPS is a perceptual metric, the reported study shows that it can be exploited as a reliable indicator of reconstruction accuracy in this case. We expect the balanced metric ${\mathscr{B}}$ to introduce an additional stability, as the PSNR and SSIM scores are sensitive to the hallucination effects^42^ which potentially may not be captured with LPIPS.

**Table 3:** The MOS survey outcome showing how often each metric was consistent with the answers (in %), stratified into SR networks trained with PROBA-V and simulated images, and interpolation.

↓ Query type	Method ↓	PSNR	SSIM	LPIPS	Total
Best acc.	SR networks (PROBA-V)	3.92	14.46	55.57	73.95
	SR networks (simulated)	11.67	0.00	7.61	19.28
	Image interpolation	1.81	4.97	0.00	6.78
	Total	17.39	19.43	63.18	100.00
Worst acc.	SR networks (PROBA-V)	5.85	0.00	0.00	5.85
	SR networks (simulated)	2.07	10.93	6.71	19.71
	Image interpolation	0.00	0.00	74.44	74.44
	Total	7.92	10.93	81.15	100.00

Overall, the reported validation has indicated that the introduced MuS2 benchmark, composed of original S-2 data coupled with HR references obtained from WV-2, can be exploited for assessing the accuracy of MISR for S-2 images. We demonstrated that the proposed evaluation protocol based on a balanced score built upon PSNR, SSIM, and LPIPS metrics is suitable for assessing the reconstruction accuracy. Based on the MOS survey, we showed that the LPIPS metric can be employed for assessing the similarity to the ground truth and that it is robust against variations resulting from acquiring images by different satellites. Additionally, we have elaborated the relevance masks which pick the regions to allow for pixel-wise evaluation performed with “traditional” PSNR and SSIM metrics.

## Usage Notes

After downloading the dataset^22^, the SR algorithms can be fed with multiple images for each scene (and for each band). Every S-2 band can be reconstructed relying on multiple images of the same band, but other bands can also be exploited during reconstruction. Also, the data can be used for SISR by picking a single LR input image for each scene and band.

To assess the quality of the super-resolved images, the code published at the Code Ocean (https://codeocean.com/capsule/8131193/tree/v2) can be exploited to compute the metrics (PSNR, SSIM, LPIPS, and the balanced score ${\mathscr{B}}$).

### Supplementary information

Supplementary Information: Mean opinion score survey

## Data Availability

The code for creating the benchmark from raw data (including the change masks and relevance masks) and for evaluating the SR outcome is available at https://codeocean.com/capsule/8131193/tree/v2. The code is documented and accompanied with usage examples.

## References

[1] Yue L (2016). Image super-resolution: The techniques, applications, and future. Signal Processing.

[2] Molini AB, Valsesia D, Fracastoro G, Magli E (2020). DeepSUM: Deep neural network for super-resolution of unregistered multitemporal images. IEEE Transactions on Geoscience and Remote Sensing.

[3] Galar M, Sesma R, Ayala C, Albizua L, Aranda C (2020). Learning super-resolution for Sentinel-2 images with real ground truth data from a reference satellite. ISPRS Annals of the Photogrammetry, Remote Sensing and Spatial Information Sciences.

[4] Razzak MT (2023). Multi-spectral multi-image super-resolution of Sentinel-2 with radiometric consistency losses and its effect on building delineation. ISPRS Journal of Photogrammetry and Remote Sensing.

[5] Drusch M (2012). Sentinel-2: ESA’s optical high-resolution mission for GMES operational services. Remote Sensing of Environment.

[6] Köhler T (2019). Toward bridging the simulated-to-real gap: Benchmarking super-resolution on real data. IEEE Transactions Pattern Analysis and Machine Intelligence.

[7] Timofte, R., Gu, S., Wu, J. & Van Gool, L. NTIRE 2018 challenge on single image super-resolution: Methods and results. In *Proc. IEEE/CVF Conference on Computer Vision and Pattern Recognition*, 852–863, 10.1109/CVPRW.2018.00130 (2018).

[8] Lanaras C, Bioucas-Dias J, Galliani S, Baltsavias E, Schindler K (2018). Super-resolution of Sentinel-2 images: Learning a globally applicable deep neural network. ISPRS Journal of Photogrammetry and Remote Sensing.

[9] Chen H (2022). Real-world single image super-resolution: A brief review. Information Fusion.

[10] Joze, H. R. V. *et al*. ImagePairs: Realistic super resolution dataset via beam splitter camera rig. In *Proc. IEEE/CVF Computer Vision and Pattern Recognition Workshops*, 518–519, 10.1109/CVPRW50498.2020.00267 (2020).

[11] Wei, P. *et al*. Component divide-and-conquer for real-world image super-resolution. In *Proc. European Conference on Computer Vision*, 101–117, 10.1007/978-3-030-58598-3_7 (Springer, 2020).

[12] Bhat, G., Danelljan, M. & Timofte, R. NTIRE 2021 challenge on burst super-resolution: Methods and results. In *Proc. IEEE/CVF Computer Vision and Pattern Recognition*, 613–626, 10.1109/CVPRW53098.2021.00073 (2021).

[13] Märtens M, Izzo D, Krzic A, Cox D (2019). Super-resolution of PROBA-V images using convolutional neural networks. Astrodynamics.

[14] Kawulok M (2020). Deep learning for multiple-image super-resolution. IEEE Geoscience and Remote Sensing Letters.

[15] Valsesia D, Magli E (2022). Permutation invariance and uncertainty in multitemporal image super-resolution. IEEE Trans. on Geoscience and Remote Sensing.

[16] An T (2022). TR-MISR: Multiimage super-resolution based on feature fusion with transformers. IEEE Journal of Selected Topics in Applied Earth Observations and Remote Sensing.

[17] Cornebise, J., Orsolic, I. & Kalaitzis, F. Open high-resolution satellite imagery: The WorldStrat dataset – with application to super-resolution. In *Proc. Conference on Neural Information Processing Systems* (2022).

[18] Beaulieu, M., Foucher, S., Haberman, D. & Stewart, C. Deep image-to-image transfer applied to resolution enhancement of Sentinel-2 images. In *Proc. IEEE International Geoscience and Remote Sensing Symposium (IGARSS)*, 2611–2614, 10.1109/IGARSS.2018.8517655 (IEEE, 2018).

[19] Zhang, R., Isola, P., Efros, A. A., Shechtman, E. & Wang, O. The unreasonable effectiveness of deep features as a perceptual metric. In *Proc. IEEE/CVF Computer Vision and Pattern Recognition*, 10.1109/CVPR.2018.00068 (2018).

[20] Wang, P., Bayram, B. & Sertel, E. A comprehensive review on deep learning based remote sensing image super-resolution methods. *Earth-Science Reviews* 104110, 10.1016/j.earscirev.2022.104110 (2022).

[21] Dong R, Zhang L, Fu H (2022). RRSGAN: Reference-based super-resolution for remote sensing image. IEEE Transactions on Geoscience and Remote Sensing.

[22] Kowaleczko P (2022). Harvard Dataverse..

[23] Tarasiewicz, T., Nalepa, J. & Kawulok, M. A graph neural network for multiple-image super-resolution. In *Proc. IEEE International Conference on Image Processing (ICIP)*, 1824–1828, 10.1109/ICIP42928.2021.9506070 (2021).

[24] Tarasiewicz, T. *et al*. Multitemporal and multispectral data fusion for super-resolution of Sentinel-2 images. *IEEE Transactions on Geoscience and Remote Sensing*10.1109/TGRS.2023.3311622. In press (2023).

[25] Deudon, M. *et al*. HighRes-net: Recursive fusion for multi-frame super-resolution of satellite imagery. *arXiv preprint arXiv:2002.06460*. 2002.06460 (2020).

[26] Salvetti F, Mazzia V, Khaliq A, Chiaberge M (2020). Multi-image super resolution of remotely sensed images using residual attention deep neural networks. Remote. Sens..

[27] Benecki P, Kawulok M, Kostrzewa D, Skonieczny L (2018). Evaluating super-resolution reconstruction of satellite images. Acta Astronautica.

[28] Prexl, J., Saha, S. & Zhu, X. X. Mitigating spatial and spectral differences for change detection using super-resolution and unsupervised learning. In *Proc. IEEE International Geoscience and Remote Sensing Symposium (IGARSS)*, 3113–3116, 10.1109/IGARSS47720.2021.9554789 (IEEE, 2021).

[29] Pomente, A., Picchiani, M. & Del Frate, F. Sentinel-2 change detection based on deep features. In *Proc. IEEE International Geoscience and Remote Sensing Symposium (IGARSS)*, 6859–6862, 10.1109/IGARSS.2018.8519195 (IEEE, 2018).

[30] Perez, D. *et al*. Combining satellite images with feature indices for improved change detection. In *Proc. IEEE Annual Ubiquitous Computing, Electronics & Mobile Communication Conference (UEMCON)*, 438–444, 10.1109/UEMCON.2018.8796538 (IEEE, 2018).

[31] Mittal A, Soundararajan R, Bovik AC (2013). Making a “completely blind” image quality analyzer. IEEE Signal Processing Letters.

[32] Venkatanath, N., Praneeth, D., Bh, M. C., Channappayya, S. S. & Medasani, S. S. Blind image quality evaluation using perception based features. In *Proc. National Conference on Communications (NCC)*, 1–6, 10.1109/NCC.2015.7084843 (IEEE, 2015).

[33] Ma C, Yang C-Y, Yang X, Yang M-H (2017). Learning a no-reference quality metric for single-image super-resolution. Computer Vision and Image Understanding.

[34] Jiang Q (2022). Single image super-resolution quality assessment: a real-world dataset, subjective studies, and an objective metric. IEEE Transactions on Image Processing.

[35] Ji, X. *et al*. Real-world super-resolution via kernel estimation and noise injection. In *Proc. IEEE/CVF Conference on Computer Vision and Pattern Recognition Workshops*, 466–467, 10.1109/CVPRW50498.2020.00241 (2020).

[36] Ziaja, M., Nalepa, J. & Kawulok, M. Data augmentation for multi-image super-resolution. In *Proc. IEEE International Geoscience and Remote Sensing Symposium (IGARSS)*, 119–122, 10.1109/IGARSS46834.2022.9884609 (IEEE, 2022).

[37] Yoo, J., Ahn, N. & Sohn, K.-A. Rethinking data augmentation for image super-resolution: A comprehensive analysis and a new strategy. In *Proc. IEEE/CVF Conference on Computer Vision and Pattern Recognition*, 8375–8384, 10.1109/CVPR42600.2020.00840 (2020).

[38] Jo, Y., Yang, S. & Kim, S. J. Investigating loss functions for extreme super-resolution. In *Proc. IEEE/CVF Conference on Computer Vision and Pattern Recognition Workshops*, 424–425, 10.1109/CVPRW50498.2020.00220 (2020).

[39] Mustafa, A., Mikhailiuk, A., Iliescu, D. A., Babbar, V. & Mantiuk, R. K. Training a task-specific image reconstruction loss. In *Proc. IEEE/CVF Winter Conference on Applications of Computer Vision*, 2319–2328, 10.1109/WACV51458.2022.00010 (2022).

[40] Kawulok, M. *et al*. On training deep networks for satellite image super-resolution. In *Proc. IEEE International Geoscience and Remote Sensing Symposium (IGARSS)*, 3125–3128, 10.1109/IGARSS.2019.8899098 (2019).

[41] Kawulok, M., Tarasiewicz, T., Nalepa, J., Tyrna, D. & Kostrzewa, D. Deep Learning for Multiple-Image Super-Resolution of Sentinel-2 Data. In *Proc. IEEE International Geoscience and Remote Sensing Symposium (IGARSS)*, 3885–3888, 10.1109/IGARSS47720.2021.9553243 (2021).

[42] Ledig C (2017). Photo-realistic single image super-resolution using a generative adversarial network. Proc. IEEE/CVF Computer Vision and Pattern Recognition.

