# Supplementary material for: A Real-World Benchmark for Sentinel-2 Multi-Image Super-Resolution
Source: Sci Data. 2023 Sep 21;10:644. doi: 10.1038/s41597-023-02538-9 (PMC10514337; doi:10.1038/s41597-023-02538-9)
Supplement: Supplementary file 1 — Supplementary Information: Mean opinion score survey [file 41597_2023_2538_MOESM1_ESM.pdf]

# A Real-World Benchmark for Sentinel-2 Multi-Image Super-Resolution

## Supplementary Information: Mean opinion score survey

**Pawel Kowaleczko[1,2], Tomasz Tarasiewicz[3], Maciej Ziaja[1], Daniel Kostrzewa[1,3], Jakub Nalepa[1,3], Przemyslaw Rokita[2], and Michal Kawulok[1,3,*]**

[1]KP Labs, Gliwice, Poland
[2]Warsaw University of Technology, Warsaw, Poland
[3]Silesian University of Technology, Faculty of Automatic Control, Electronics and Computer Science, Gliwice, Poland
[*]corresponding author(s): Michal Kawulok (michal.kawulok@polsl.pl)

**Table S1.** Questionnaire sent to the respondents to determine the mean opinion score (MOS). The questions and images are in the same order as in the survey. There are 15 questions concerned with the reconstruction accuracy. For each image, we provide the metric used to determine the best (or worst) match alongside the method used to obtain that result (obviously, such information was not revealed to the respondents).

1. Which of the following images presents the urban area in the MOST DETAILED WAY?

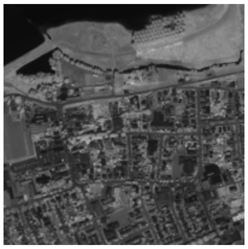

Reference image

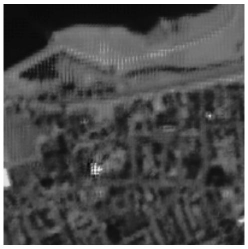

PSNR (HRN_SIM), 21 votes

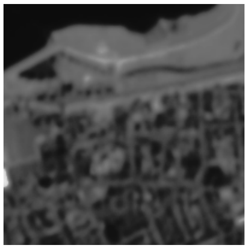

SSIM (Lanczos), 9 votes

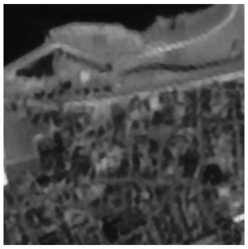

LPIPS (RAMS_NIR), 136 votes

2. Which of the following images is the MOST DIFFERENT from the reference image?

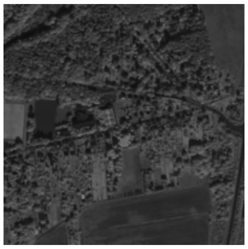

Reference image

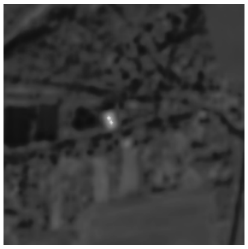

LPIPS (Bicubic), 119 votes

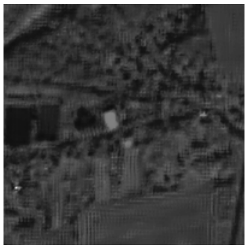

SSIM (HRN_SIM), 29 votes

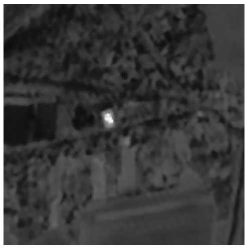

PSNR (HRN_NIR), 18 votes

3. Which of the following images presents the annotated woodless area in the MOST ACCURATE way?
This area was annotated in red in the reference image (right).

| Reference image | | | |
|---|---|---|---|
| 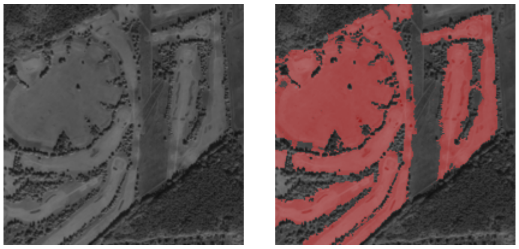 | 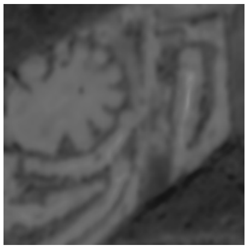 | 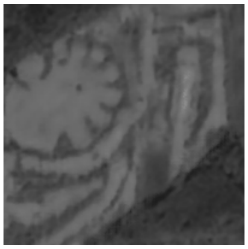 | 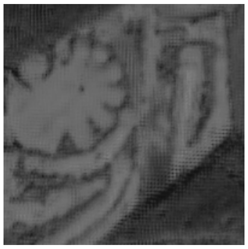 |
| Reference image | SSIM (Lanczos), 10 votes | LPIPS (HRN_NIR), 89 votes | PSNR (RAMS_SIM), 67 votes |

4. Which of the following images presents the SHAPE of annotated buildings in the WORST way?
The buildings of interest are annotated in red in the reference image.

| | | | |
|---|---|---|---|
| 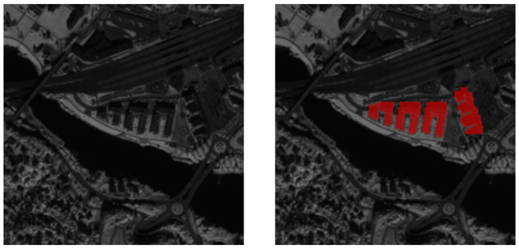 | 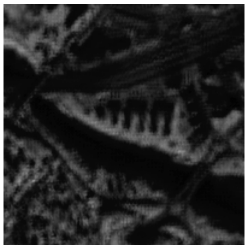 | 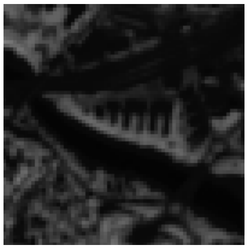 | 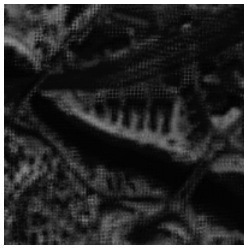 |
| Reference image | PSNR (HRN_SIM), 10 votes | LPIPS (NN), 139 votes | SSIM (RAMS_SIM), 17 votes |

5. In which image the rural roads are presented in the CLEAREST (MOST DETAILED) way?
The roads of interest are annotated in red in the reference image.

| | | | |
|---|---|---|---|
| 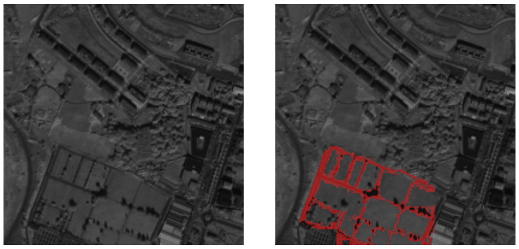 | 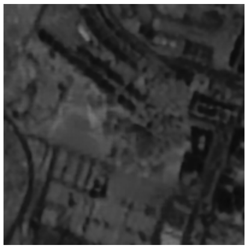 | 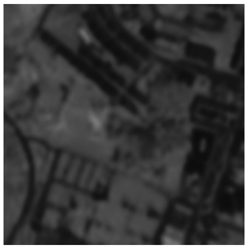 | 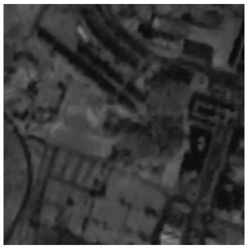 |
| Reference image | LPIPS (HRN_NIR), 69 votes | PSNR (Lanczos), 21 votes | SSIM (RAMS_NIR), 76 votes |

6. In which image the buildings manifest THE LEAST LEVEL OF DETAIL?

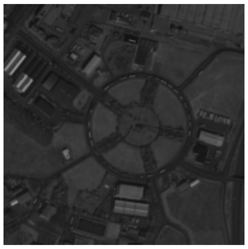
Reference image

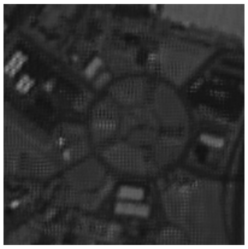
LPIPS (RAMS_SIM), 78 votes

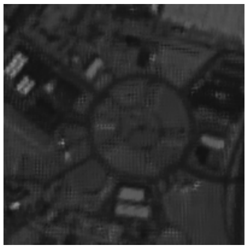
SSIM (HRN_SIM), 50 votes

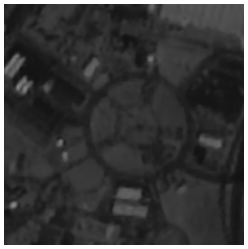
PSNR (HRN_NIR), 38 votes

7. In which of the following images the junction is presented in the MOST ACCURATE (DETAILED) way?

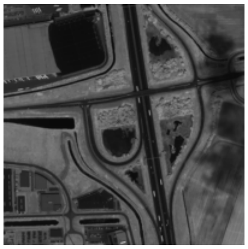
Reference image

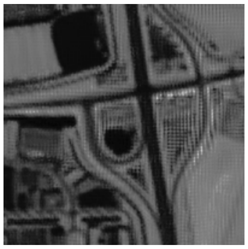
PSNR (RAMS_SIM), 30 votes

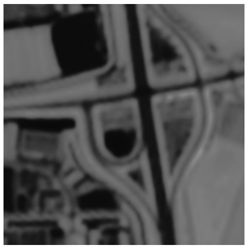
SSIM (Lanczos), 34 votes

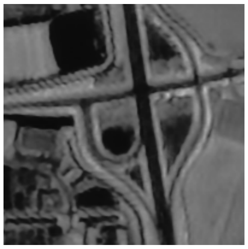
LPIPS (RAMS_NIR), 102 votes

8. In which of the following images it is the MOST CHALLENGING to distinguish separate trees?

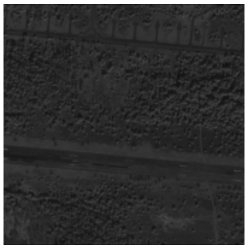
Reference image

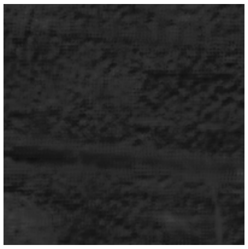
SSIM (RAMS_SIM), 12 votes

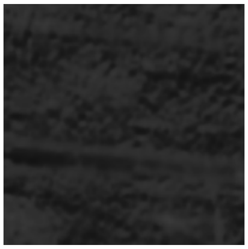
LPIPS (Bicubic), 150 votes

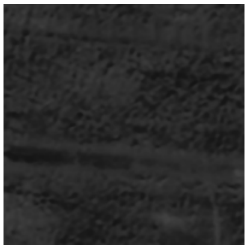
PSNR (RAMS_NIR), 4 votes

9. In which of the following images the area of interest (annotated in red in the Reference image) is presented in the MOST DETAILED way?

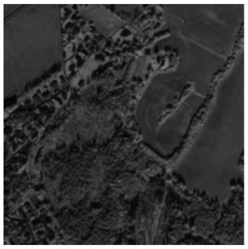
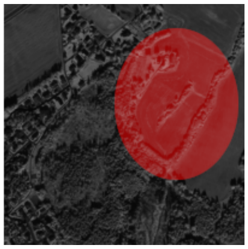
Reference image

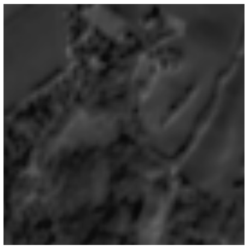
PSNR (Linear), 3 votes

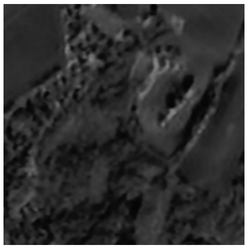
LPIPS (RAMS_NIR), 150 votes

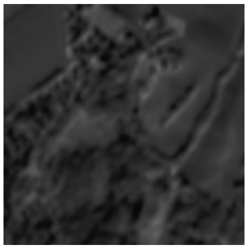
SSIM (Bicubic), 13 votes

10. Which of the following images presents the coastal area in the LEAST ACCURATE (LEAST DETAILED) way? The area of interest is rendered in red in the reference image.

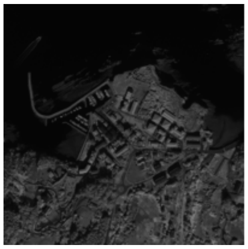
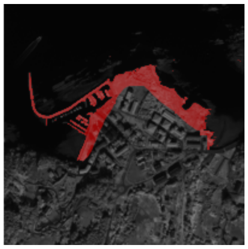

Reference image

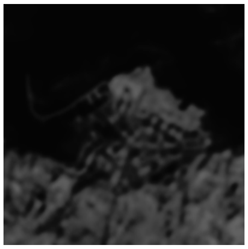

LPIPS (Lanczos), 154 votes

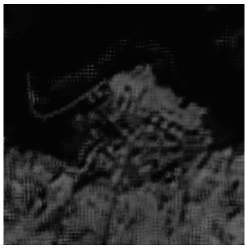

SSIM (RAMS_SIM), 2 votes

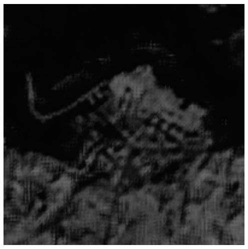

PSNR (HRN_SIM), 10 votes

11. Which of the following images presents the roads in the MOST DETAILED way? The roads of interest are annotated in red in the reference image.

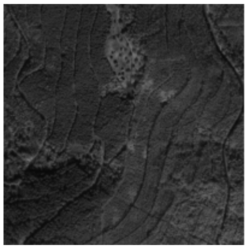
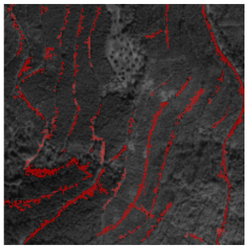

Reference image

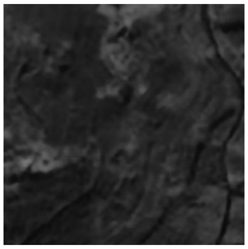

SSIM (HRN_RED), 10 votes

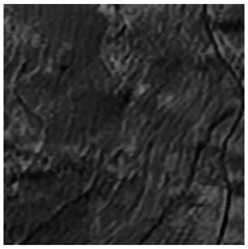

LPIPS (RAMS_NIR), 126 votes

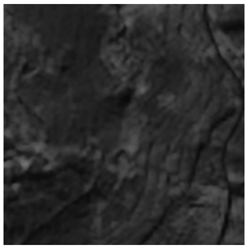

PSNR (RAMS_RED), 30 votes

12. In which of the following images the urban area is presented in the LEAST DETAILED way?

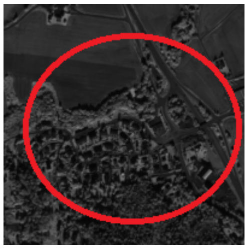

Reference image

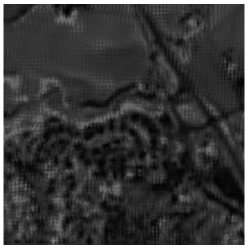

SSIM (RAMS_SIM), 11 votes

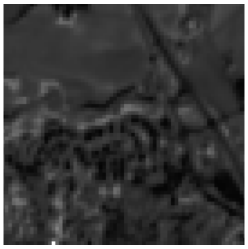

LPIPS (NN), 151 votes

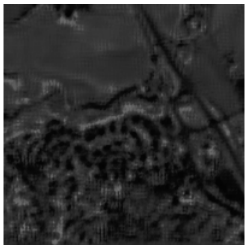

PSNR (HRN_SIM), 4 votes

13. Which of the following images allows one to count the buildings in THE MOST CONVENIENT way?
The buildings of interest are annotated in red in the reference image.

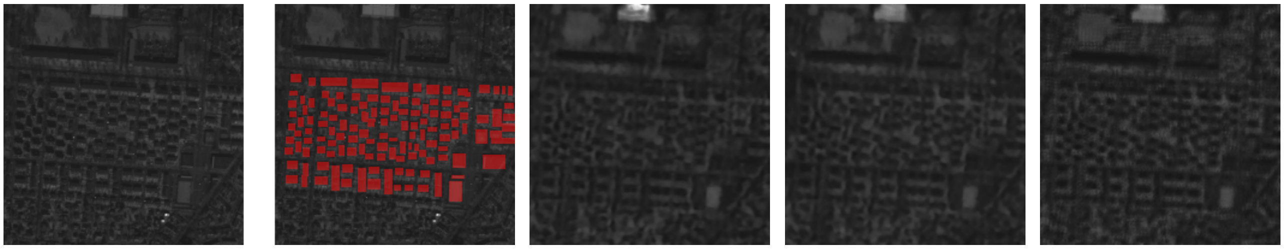

Reference image | PSNR (RAMS_NIR), 22 votes | SSIM (HRN_NIR), 43 votes | LPIPS (HRN_SIM), 101 votes

14. In which of the following images the parking is presented in the LEAST DETAILED way?
The parking of interest is annotated in red in the reference image.

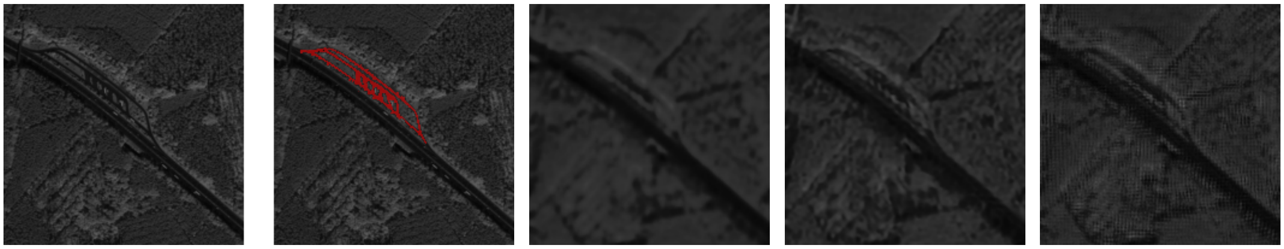

Reference image | LPIPS (Bicubic), 152 votes | PSNR (RAMS_NIR), 8 votes | SSIM (RAMS_SIM), 6 votes

15. Which of the following images presents the roads of interest MOST FAITHFULLY?
The roads of interest are annotated in red in the reference image.

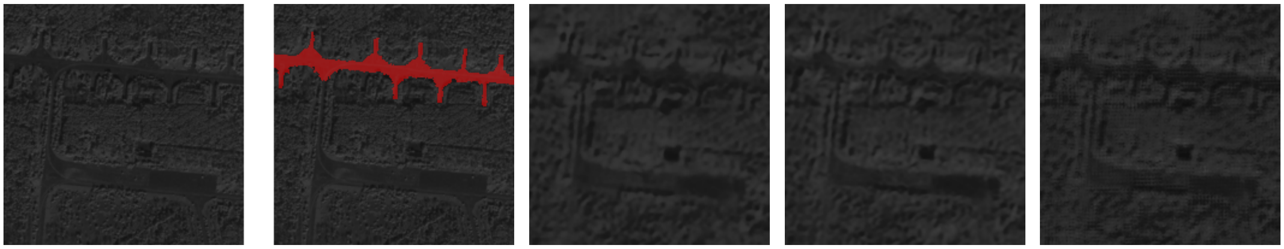

Reference image | SSIM (HRN_RED), 63 votes | LPIPS (RAMS_NIR), 66 votes | PSNR (RAMS_SIM), 37 votes
